# Predicting the Performance of Students Using Deep Ensemble Learning

**DOI:** 10.3390/jintelligence12120124

**Published:** 2024-12-03

**Authors:** Bo Tang, Senlin Li, Changhua Zhao

**Affiliations:** School of Computer and Artificial Intelligence, Huaihua University, Huaihua 418000, China; lsl@hhtc.edu.cn (S.L.); lala_zhao@163.com (C.Z.)

**Keywords:** student performance prediction, machine learning, deep belief network, particle swarm optimization

## Abstract

Universities and schools rely heavily on the ability to forecast student performance, as it enables them to develop efficient strategies for enhancing academic results and averting student attrition. The automation of processes and the management of large datasets generated by technology-enhanced learning tools can facilitate the analysis and processing of these data, which provides crucial insights into the knowledge of students and their engagement with academic endeavors. The method under consideration aims to forecast the academic achievement of students through an ensemble of deep neural networks. The proposed method presents a new feature-ranking mechanism based on existing approaches. This mechanism is effective in identifying the most pertinent features and their correlation with the academic performance of students. The proposed method employs an optimization strategy to concurrently configure and train the deep neural networks within our ensemble system. Furthermore, the proposed ensemble model uses weighted voting among its learning components for more accurate prediction. Put simply, the suggested approach enhances the accuracy of academic performance predictions for students not only by employing weighted ensemble techniques, but also by optimizing the parameters of deep learning models. These experimental outcomes provide evidence that the proposed method outperformed the alternative approaches, accurately predicting student performance with a root-mean-square error (RMSE) value of 1.66, a Mean Absolute Percentage Error (MAPE) value of 9.75, and an R-squared value of 0.7430. These results show a significant improvement compared to the null model (RMSE = 4.05, MAPE = 24.89, and R-squared = 0.2897) and prove the efficiency of the techniques employed in the proposed method.

## 1. Introduction

Education is vital to the development of a country. It is also a crucial tool for achieving success in one’s existence. Every academic establishment endeavors to provide its students with a high-quality education with the intention of enhancing their learning experience (Ramaphosa et al. 2018). In light of the COVID-19 pandemic, which halted the operations of conventional educational institutions, the rapid development of technology and the pervasive adoption of technology-assisted educational platforms ushered in new paradigms for the education system (Mustapha et al. 2021). These platforms possess the capability to observe the actions of students and collect data, which can subsequently be employed to analyze issues faced by students and implement suitable remedies in real-time (Romero and Ventura 2020). Implementing preventive and remedial measures could potentially enhance the performance of academic institutions while simultaneously reducing the probability of student failure. Educational data mining (EDM) provides explicit solutions to these challenges, which are advantageous for instructors and learners alike. Educational data mining (EDM) is an emerging field of study that analyzes data from academic sources for various purposes. One of the most widely used implementations of EDM is the prediction of student academic performance. It has been acknowledged that the analysis and interpretation of student performance is the most important issue in academia, requiring appropriate assessment, evaluation, and analysis techniques. In the contemporary economy, which is predicated on knowledge, pupils contribute significantly to the socioeconomic progress of a nation. Thus, maintaining students’ academic progress is critical (Hernández et al. 2020). A variety of learning approaches founded on Information and Communication Technology (ICT) have been implemented by higher education institutions (HEIs). Multiple learning environments are utilized in these strategies to facilitate the learning process and efficiently transfer knowledge to pupils (Palomo-Duarte et al. 2014). In addition, for auditing and recovery purposes, these environments keep logs of user interactions and interactions with the environment (Sarwat et al. 2022).

Developing a more precise student model that can automatically gather data about students is a prerequisite for developing an effective adaptive education system. As a result, one of the best techniques for determining a learner’s cognitive level is knowledge tracing (KT) (Corbett and Anderson 1994; Baker et al. 2008; Pelánek 2017). To measure student knowledge and predict future performance, many techniques based on machine learning classifiers have been extensively used in this field. The Performance Factor Analysis (PFA) method has been shown via experiments in various research articles to perform better than several other KT techniques (Gong et al. 2011; González-Brenes et al. 2014; Xiong et al. 2016; Minn et al. 2018). However, the overwhelming desire to enhance the knowledge tracing models that are now in use prompts researchers to suggest intricate expansions in an effort to provide prediction outputs that are more accurate. All of these enhancements, nevertheless, have been improved almost completely in an educational setting. To be more precise, in order to make changes, researchers have solely examined the impact of a subset of students’ behavior throughout the learning process (Asselman et al. 2023). Although PFA has given a framework for the assessment of student performance, more recent studies have focused on various aspects of performance determinants. This study includes these advancements by exploring the use of socio-economic status, learning preferences, and technology incorporation. These broaden the view and facilitate a deeper investigation of student performance prediction.

Many applications have made use of artificial intelligence (AI) and machine learning, such as text translation, audio recognition, natural language processing, image classification, and educational data mining. EDM is concerned with analyzing and evaluating different aspects of educational datasets gathered from various e-learning environments or higher education institutions using a variety of data mining techniques, including classification, regression, time series analysis, and association rule mining. EDM is a widely used method for building predictive models via the extraction of significant data and hidden patterns that might be used for teaching and learning (Mengash 2020). By predicting students’ future academic performance, machine learning can facilitate the early identification of students at risk, enabling targeted interventions to enhance learning outcomes and potentially improve grades (Sapare and Beelagi 2021). Three major advances are introduced by the suggested strategy. First, it selects attributes that are relevant to students’ performance by ranking the student attributes according to their importance, and then selecting the most significant ones. Second, it integrates the particle swarm optimization (PSO) technique for deep belief neural network optimization and weight setting across different ensemble systems. Finally, it uses weighted averaging to refine the final weights of each DBN model in the ensemble system using autoencoder models. Among the procedures used in our research study are the following:

- Introducing an innovative method for predicting students’ academic performance using deep ensemble learning.
- Enhancing precision in forecasting students’ academic performance through the amalgamation of deep learning models, optimization, and reinforcement learning.
- Proposing a comprehensive and in-depth approach to predicting students’ academic performance across various domains by combining diverse learning models with different configurations.

This paper is structured as follows: The second section analyzes comparable works. The proposed methodology is explained in Section 3. The outcomes of its execution are presented in Section 4. Finally, the discussion is presented and the conclusions are drawn in Section 5 and Section 6.

## 2. Related Work

In this section, we examined previous studies on predicting students’ academic performance using a variety of techniques.

By integrating clustering and prediction methodologies, Francis and Babu (2019) proposed a predictive strategy for students’ academic performance. In order to generate predictions, they utilized decision trees (DTs) (Priyanka and Kumar 2020), neural networks (Asadollahfardi 2015), naive Bayes (NB) (Wickramasinghe and Kalutarage 2021), and Support Vector Machines (SVMs) (Pisner and Schnyer 2020) as classifiers to evaluate the demographic, academic, and behavioral characteristics of the student dataset. By achieving an average accuracy of 0.7547 on features of the student dataset, the results obtained from the proposed hybrid technique unequivocally indicate that clustering exhibited superior performance compared to alternative classifiers. However, the study is limited by the small sample size and potential overfitting of the clustering-based approach.

As a neural network, Bendangnuksung (2018) suggested a deep neural network (DNN) for classifying and predicting student performance. Establishments would profit from having forewarnings regarding their failing pupils in order to promptly offer them assistance. Their study utilized an educational dataset consisting of 500 students. This dataset, referred to as xAPI Edu Data on Kaggle, was made public. The dataset was collected using Kalboard 360, a learning management system. DNN achieved an accuracy of 84.3% despite utilizing a reduced amount of data, significantly surpassing alternative machine learning prediction methodologies. DNNs are capable of generating even more impressive outcomes when presented with larger datasets. Their ability to learn complex, non-linear patterns and relationships within the data enables them to extract valuable insights from large datasets (Ahmed et al. 2023). This is particularly advantageous in domains with abundant data, where large-scale datasets can provide a rich source of information for modeling. For example, researchers in (Gütter et al. 2022), demonstrated that increasing the dataset size for training a DNN could improve the robustness of the DNN against noise.

During the spring semester of a private college in Oman, Hasan et al. (2018) proposed a methodology for predicting the academic achievement of 22 students by utilizing DT algorithms and academic data. The DT techniques were evaluated in order to predict Moodle access time and performance, utilizing the data mining tool WEKA. In terms of accuracy, DT outperforms alternative classification algorithms, as demonstrated by the outcomes. The proposed methodology evaluates the efficacy of the module and contributes to the enhancement of student grades. However, the size of studied population in this study is very limited and makes the generalizability of the research findings questionable.

A study on the prediction of students’ academic performance and quality of life utilizing SVM was conducted by Raihana and Farah Nabilah (2018). The information utilized in their inquiry was submitted by Bachelor of Science candidates. Quality of life was evaluated through the utilization of a questionnaire that encompasses various dimensions, such as social relationships, environment, physical and psychological well-being, and overall quality of life. Utilizing a student’s CGPA as an indicator of academic achievement, SVM algorithms were utilized to classify students according to the assessment variables provided. The proposed methodology yielded a model accuracy of 73.33% on average. However, the research was limited by the focus on Bachelor of Science candidates and the potential for bias in the quality of life questionnaire.

An academic achievement prediction framework for students, proposed by Divyabharathi and Someswari (2018), makes use of the NB prediction approach. To perform the analysis for their research, they utilized a dataset comprising the anonymous records of 500 pupils, obtained from the database of an educational institution. They utilized the NB algorithm to predict students’ achievement in specific subjects through an analysis of their previous examination outcomes. They accomplished their objective with an exceptional level of precision, 94%. The study relied solely on the naive Bayes algorithm, limiting its ability to capture complex relationships.

Zorić (2019) examine performance perditions utilizing 76 students from the University of Applied Sciences Baltazar in Zaprešić. In their research, prediction was performed on the basis of the economic and demographic attributes of the students. Also, grade point average was considered the target variable in the study. Due to the scarcity of data regarding students in lesser grades (only 8%), the author achieved a prediction rate of 93.42% through the implementation of neural networks. The research was limited by the small dataset, particularly for lower-grade students, and the potential for bias in the economic and demographic attributes used for prediction.

A prediction model for the study of student attrition was developed by Gamao and Gerardo (2019) through the integration of the modified Mutated Firefly Algorithm (MMFA) with relevant categorization algorithms such as NB and DT, utilizing the cumulative records of the students. Through the utilization of the mutation process and firefly behavior, the MMFA successfully traversed the search space in pursuit of the optimal solution or model. Hence, in an effort to mitigate attrition rates, academic administrators at Davao del Norte State College might employ the algorithm’s precision in formulating academic regulations that enhance student performance across subjects and extracurricular engagements. Further investigation into the potential applications of the MMFA could be undertaken by future researchers through its integration with other relevant categorization algorithms. Additionally, the study was limited by focusing on predicting student attrition rather than overall academic performance.

As expected by Gil et al. (2020), the reasons for student dropout pose a major and challenge for educators and institutions. They therefore looked into whether using data mining methods may help each institution solve this issue, wherein the student dropout indicators were correctly predicted by the data mining classification technique. The most widely used data mining approaches were used to identify the student dropout indicators, based on C4.5 and NB. These two different classification algorithms were trained and assessed using the ten-fold cross-validation technique. It alerts the instructor, who may then take necessary action to improve the pupils’ performance by giving them specialized coaching and counseling. The study was limited by the potential for bias in the data mining techniques used and the need for further validation in different educational contexts.

Singh and Pal (2020) proposed a variety of machine learning approaches to evaluate the performance of students. Five machine learning algorithms were employed to classify pupil predictions: Extra Tree (ET), the Passive Aggressive Classifier (PAC), SVM, Linear Discriminant Analysis (LDA), and the Radius Neighbor Classifier (RNC). Among these various approaches, SVM had the highest accuracy at 94.86%. The second-highest level of accuracy, achieved for LDA, was 93.21%. Regarding the corpus of research concerning the prediction of pupil performance, their accuracy was the highest. By utilizing the initial-stage prediction as a feature rather than conducting a distinct training process, the approach based on machine learning reduced generation errors and accumulated substantially more data. The predictions made by this model relied solely on an SVM predictor, limiting its ability to capture complex relationships.

Waheed et al. (2020) implemented a deep artificial neural network to identify at-risk pupils by analyzing clickstream data from virtual learning environments and identifying them based on specific characteristics. In contrast to logistic regression and SVM models, the proposed model achieved a classification accuracy ranging from 84% to 93%. In addition, its performance exhibited a significant improvement over that of SVMs (79.95–89.14%) and logistic regression (79.82–85.60%). Previous studies’ findings were consistent with the substantial impact that the model gained from the incorporation of legacy and assessment-related data. The study focused on clickstream data from virtual learning environments, which might not be applicable to traditional classroom settings.

To forecast the academic achievement of college students, Tsiakmaki et al. (2020) examined the efficacy of transfer learning derived from deep neural networks. By analyzing data from five required courses in two undergraduate programs, the research demonstrated that transfer learning strategies could effectively identify individuals prone to failure, provided that correlated course datasets, which are increasingly available in many educational institutions, are accessible. However, the effectiveness of transfer learning depends on the availability of correlated course datasets, which might not be available in all institutions.

Beckham et al. (2023) identified characteristics that might have impacted student performance through the use of Pearson correlations. The results of the study indicated that previous academic shortcomings negatively affected student grades (−0.360415), while the education of the mother had a positive effect (0.217147). Predicting student grades with machine learning models, the MLP 12-Neuron model performed the best in terms of root-mean-square error (RMSE). Given the large and increasing student population, providing individualized interventions to address academic challenges for each student is impractical. The study relied solely on Pearson correlations for feature selection, which might overlook non-linear relationships.

Lau et al. (2019) predicted student performance by employing neural network modeling and statistical analysis. Their model comprised eleven input factors, two layers of hidden neurons, and one output layer. The Levenberg–Marquardt algorithm was implemented as the training rule for backpropagation. The Levenberg–Marquardt algorithm is an iterative method for determining the minimum of a function written as the sum of squares of non-linear functions. Finding the minimum of a function defined as the sum of squares of non-linear functions is performed iteratively using the Levenberg–Marquardt algorithm (Yu and Wilamowski 2018). Also, the technique of backpropagation (Asaad and Ali 2019) involves examining the error ratio from the preceding iteration in order to modify the weights of a neural network. The accuracy of the model is 84.8%. The study used a single neural network model, limiting its ability to handle complex relationships.

By evaluating student performance data with data mining techniques, including Nave Bayes, ID3, C4.5, and SVM, Pallathadka et al. (2023) assisted academic institutions in reducing failure rates and enhancing student performance. The experimental investigations utilized the UCI machinery student performance dataset to evaluate the accuracy and error rate metrics of the algorithms. The study demonstrated the potential of these techniques for predicting student performance. However, the study did not explore deep learning methods, which might offer better performance for complex tasks.

Using video learning analytics and data mining techniques, Hasan et al. (2020) predicted the performance of 772 students enrolled in e-commerce and e-commerce technologies courses at a higher education institution (HEI). The 88.3% accuracy with which the Random Forest method predicted successful pupils demonstrates the efficacy of these strategies. The study focused on video learning analytics, which might not be applicable to all learning environments.

Alyahyan and Düştegör (2020) devised a methodical approach for educators to forecast student achievement utilizing machine learning techniques. Among the options, criteria, and strategies covered in the manual were the following: defining success, focusing on student characteristics, and selecting the most effective strategy. This facilitated the provision of data mining tools to educators, thereby empowering them to actualize their capabilities to the greatest degree possible within the realm of education. The study provided general guidelines for educators rather than a specific predictive model.

Gimenez et al. (2018) used performance data in an attempt to establish a connection between students and the social development of the districts in Costa Rica. The study intended to establish the effects of social factors on academic performance using PISA 2012 data and a composite social development index. The study was limited by focusing on the relationship between social factors and academic performance, rather than predicting individual student performance.

In a cross-sectional study, Antonopoulou et al. (2020) compared the Mediterranean diet scores, achievement scores, and depressive symptoms in university students. The study also used a literature review to determine the trends in the dietary patterns and their effects on students based on the existing literature. However, the study has the limitation of not being able to establish the causal relationships between variables.

Agnafors et al. (2021) examined the temporal cross-sectional association between mental health and academic achievement from childhood to young adulthood. With data from a birth cohort, the study aimed to establish when and how mental health problems affect education. The study focused on the relationship between mental health and academic achievement, rather than predicting individual student performance.

Bruffaerts et al. (2018) examined a cross-sectional survey to determine the proportion of first-year students who had mental health disorders and how these affected their performance. Mental health disorders and their effects on GPA were explored in the study to determine the effects of mental health on functioning. This research also focused on the impact of mental health disorders on performance, rather than predicting overall academic achievement.

Previous research has greatly contributed to the prediction of student performance, but these studies are sometimes characterized by small sample size, single model usage, and no feature selection. Some of these limitations can affect the degree of reliability or indeed the validity of some of the predictions made. To fill these gaps, our research presents a new ensemble method that includes feature ranking and weight optimization. In this paper, we propose an ensemble of multiple deep neural networks and select the most appropriate features in order to enhance the model’s performance and its resilience. Also, the weight optimization strategy makes it possible to assign the right weight to each model’s contribution towards the final prediction, which is accurate. Therefore, the present study extends previous research by overcoming its shortcomings and providing a better and more efficient way to predict student performance.

## 3. Research Method

This section provides an overview of the dataset prior to discussing the proposed approach for forecasting student performance through the integration of reinforcement learning and deep learning models. The suggested method was implemented using the MATLAB 2020a software.

### 3.1. Data Collection

The data utilized in this study were gathered via the dissemination of questionnaires to students enrolled in higher education institutions in Nanjing, China. The information contained in this database was provided by 628 students enrolled in two technical and engineering faculties. The educational conditions of the students were gathered during the distribution of these questionnaires at the commencement of the course. Subsequently, at the conclusion of the academic term, the mean scores of all responding students were collected as the dependent variable. A total of 383 records in the gathered dataset pertained to female students, while the remaining records pertained to male students. The mean age of the respondents was 23.78 years, spanning a range of 20 to 31 years. The inventory of data accessible through the database is detailed in Table 1.

The dataset consists of thirty independent variables that characterize the students’ life, academic environment, and socioeconomic status. These variables were chosen based on prior theoretical and empirical research which has shown that they have a strong relationship with student performance. For example, Bendangnuksung (2018) and Raihana and Farah Nabilah (2018), as well as Divyabharathi and Someswari (2018), have shown that academic performance is significantly predicted by the parents’ education level, family relationships, and study habits. These factors could act as incentives or disincentives to students, affect their ability to obtain learning resources, and modulate their learning environment, thus impacting their performance. We include these variables in the model as we would like to include all the parameters that describe students and their situations which could impact the results. It should be noted that all independent variables, I1-I30, were self-reported by the students participating in the study using a paper-and-pencil test. Additionally, the dependent variable of students’ final average grades was not self-reported and was obtained through the institution. The dependent variable of the dataset is the students’ ultimate average scores, which serve as an indicator of their academic accomplishments. The age attribute is represented by a natural number denoted in years. The attributes “weekly study time”, “distance from home to school”, and “amount of free time after classes” are quantified in minutes. The residence status attribute specifies whether the pupil resides with their parents, in a dormitory, or in an autonomous home. The educational attainment of the parents is denoted by a numeric value ranging from zero to four, with the following values assigned: illiterate, primary, guidance, high school, and higher education. The attribute “I18: Use of Extracurricular Classes” refers to the student’s participation in extracurricular or optional classes. These classes include either academic enrichment programs, tutoring sessions, or other forms of additional instruction. In contrast, the attribute “I19: Participation in Extracurricular Activities” refers to the student’s participation in activities outside of regular coursework, such as sports, clubs, or volunteer work. It is valued as yes if the student participated in at least one extracurricular or optional class, and otherwise, this attribute is valued as no. The employment status of parents is classified into one of the following categories: domestic, medical, or services. One of the following values may influence the selection of a study location: proximity, credibility, appropriate field of study, and so on. The lawful guardian attribute specifies either the parent, father, or another individual. The values assigned to the level of relationship with family members and current health status are described on a Likert scale, with five points ranging from zero (indicating the worst possible) to four (indicating the best possible). Additional nominal database features consist of logical attributes that accept either true or false values. The dataset’s target parameter is the student’s final average score, denoted as a numeric value ranging from 0 to 20.

### 3.2. Proposed Method

**Figure 1:**
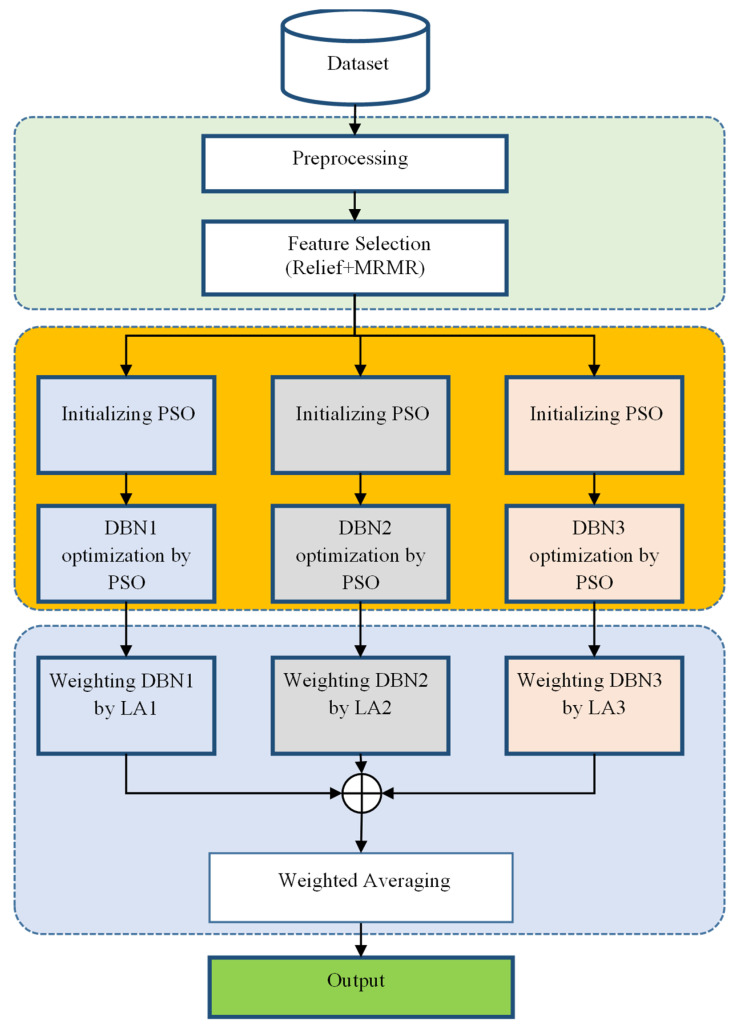
A diagram of the proposed method.

Utilizing a general mechanism utilized in prior research, the proposed method for predicting the academic performance of students seeks to enhance the overall performance of the system in prediction by providing suggestions for enhancing each stage. The method under consideration can be delineated into the following primary stages (Figure 1):

1. Data preparation and feature selection;
2. Constructing an ensemble model of the DBN;
3. Weighting learning components in the ensemble system based on reinforcement learning and prediction.

The proposed method commences with the preprocessing of the dataset samples in the initial phase. Following the process of replacing absent values and converting nominal characteristics to numeric values, a formatted set of data records is produced. A combined approach utilizing the Relief and MRMR feature-ranking methods is implemented subsequent to the initial phase of the proposed method in order to identify the most pertinent features that pertain to the academic performance of students. The chosen characteristics are employed to train a collection of DBNs, which, when combined, constitute the ensemble model that is proposed for the purpose of predicting the target variable. The PSO algorithm is entrusted with the responsibility of training and modifying the optimal weight vector for every DBN in this ensemble system. The PSO algorithm was used for this purpose as it is a powerful algorithm to solve non-linear optimization problems. Due to its population-based nature, it is possible to traverse the solution space and find the optimal or near-optimal weight configurations. Moreover, PSO can maintain the exploration and exploitation of the solution space well, so it is suitable for our case, in which we need to find suitable weights for multiple DBN models.

Once the DBN models have been configured and trained, learning automaton (LA) models are employed to modify the weight values of each model and ascertain its influence on the ensemble system’s output. This approach involves the LA models modifying the weight values of the DBN models in accordance with their training error, with the objective of reducing the ensemble system’s overall error. Once the weight values of the learning components have been determined, the models that have been trained and weighted are applied to forecast the target variable in fresh samples. To achieve this, the sum of the obtained values for the deep models is divided by the sum of the weight values, and the output of each model is multiplied by its corresponding weight. The following section provides additional information regarding the proposed methodology.

#### 3.2.1. Preprocessing and Feature Selection

As the initial stage of the proposed model, data preprocessing is utilized to prepare the database for subsequent processing. The procedure of data preprocessing consists of the following two stages:

(a) Converting nominal features to numerical: The nominal elements comprising the database are classified into ordinal and discrete categories. For instance, we commence by extracting a list containing every nominal value associated with each of these features. In the case of discrete nominal features, the ascending order in which the value appears most frequently is utilized to generate the list. Conversely, the ordinal nominal feature values are arranged in ascending order according to their rank, as determined by their respective values in the list (for example, {never = 1, rarely = 2, sometimes = 3, often = 4, always = 5} or {no = 1, yes = 2}). Following the creation of the sorted list of nominal values, each nominal value is assigned its corresponding position number in the sorted list. The resultant list contains the value in lieu of the location number of each value after sorting. By means of this vectorization procedure, every record is transformed into a numeric vector.

(b) Managing records with missing values: The dataset includes three records including missing values. In the following step, we apply different techniques to both nominal and numerical features. As shown in Gunn et al. (2023) and Nijman et al. (2022), machine learning is an efficient approach for managing records with missing values. Accordingly, for the numerical features, we use k-nearest neighbors with K = 5 to replace the missing values. The appropriate value of K is chosen experimentally. In this approach, the Euclidean distance between the record with missing values and other dataset instances is measured. In this process, only records without missing values are considered for measuring distance. Then, the K-closest instances are chosen and the missing value is replaced with the average values of the attribute in the selected instances. The same procedure is applied for the ordinal nominal feature. In contrast, each discrete value for the absence of nominal features is replaced with zero.

After the features have been preprocessed, a feature selection process is carried out to determine which characteristics have the most impact on students’ academic achievement. In the first phase, known as feature selection, the database characteristics indicated in Table 1 are simultaneously rated using two distinct algorithms. Relief is the top-ranked approach. Relief is an algorithm that uses filters to identify the most notable characteristics in the training data and then ranks them based on their importance. The ideal approach for calculating relief involves first calculating the distance between each sample and all other samples. This distance is then used to compute the weight of each characteristic for each sample. In this case, the weight is determined by assigning a greater weight value to the characteristics that are closest to the other samples. As a result, each feature’s weight value represents its relative relevance. In contrast, the Relief method takes into account the characteristics’ relative significance and evaluates each feature repeatedly. Relief’s computational process is described in depth in Urbanowicz et al. (2018).

MRMR is the second approach that is included in the suggested procedure. By taking into account both the data redundancy and the features’ relationships with the target variable, this strategy explores the significance of features. The way this strategy works is by first determining how each characteristic correlates with the target variable. It is quantified using a variety of indicators, such as the correlation coefficient, which is used in the suggested technique, or mutual information. The weight of the information that each characteristic provides may then be quantified by calculating its relationship with the other features. Additionally, the two computed criteria are used to generate the relationship scores for each feature. This algorithm’s computational specifics are given in Jo et al. (2019), and its recurrence is ignored. Following the computation of each feature’s relevance based on the MRMR and Relief methods, the results are as follows:

(1) $$W_{f}=\frac{1}{2}\times \left(\frac{M_{f}}{\sum_{i=1}^{N}M_{i}}+\frac{R_{f}}{\sum_{i=1}^{N}R_{i}}\right)$$

where *M_f_* and *R_f_*, respectively, stand for the weight values that the Relief and MRMR methods found for feature *f*. *N* also indicates how many input characteristics there are in total. The Backward Elimination Feature Selection (BEFS) (Foli 2018) technique is used to eliminate extraneous features at the very end of the feature selection process. In BEFS, initially, the model is trained using all of the candidate features that were chosen as a collection of relevant features, and the training error is calculated by running the model with all of the candidate features. After this, the procedure would become iterative, progressively eliminating each feature, beginning with the least significant. After this, we train the learning model on the remaining features while removing the feature from the model that performed the poorest out of all the chosen features. Next, the learning model’s training error is assessed using the shrunk dimensions. The steps of feature removal, model training using the set, and the evaluation of training error are repeated if the new set reduces the model’s training error. If not, the feature removal process is terminated, and the set of features with the lowest training error is deemed to contain the relevant features. The second step of the suggested technique then uses the selected characteristics as its input.

#### 3.2.2. Constructing an Ensemble Model of DBNs

The ensemble model used in the suggested approach is composed of DBN models, which have a dynamic structure and may be configured in this way to improve prediction accuracy. Three dynamic DBN models, each trained on various data subsets, form the foundation of this integrated system. Our approach entails using the PSO algorithm to tackle the structure and weight optimization issue for every DBN. Due to these factors, the training function and topology of the DBNs used in the disclosed ensemble system to predict students’ academic performance are not stated; instead, the PSO algorithm decides both of them. Because of this, each DBN model in this step of the suggested technique has a particle swarm optimizer whose job it is to determine the optimal topology and weight vector for each DBN based on its performance, as determined by the training error criteria (as its fitness function) (Guan 2023). A detailed explanation of PSO’s optimization phases can be found in Gad (2022). Our model uses this technique to optimize every DBN model. As a result, this portion of the algorithm simply shows the fitness function, the particle structure (solution vector), and the PSO algorithm that is used in the suggested technique. In a similar manner, the PSO is used to optimize every DBN model individually.

The suggested technique defines the topology of the DBN, weights, and biases of the connections using the solution vector of the PSO algorithm. The solution vector is connected to the two components of the optimization method as it consists of two related jobs. The structure of the network is described in the first part of the solution vector, and the weights and biases of the neurons (given the structure defined in the first part of the solution vector) are calculated in the second portion. Therefore, the topological analysis that was performed for the DBN determines the variable length of the solution vectors in the PSO method. Given that the DBN has an unlimited number of topological states, the model has to take certain constraints into account for the network topology portion of the solution vector. For the first component of the solution vector, two primary limits are taken into consideration in order to restrict the search space:

- First of all, the number of hidden layers in each DBN should be at least equal to 1 and not more than 3. To this end, the first segment of the solution vector consists of 1 to 3 elements, and the number in each of these elements of the first part of the solution vector indicates the number of neurons required for each layer.
- As a second point, every hidden layer in the DBN has a number of neurons ranging from 2 to 10. Henceforth, every element in the solution vector component number belongs to the natural numbers in the range of 2 to 10.

**Figure 2:**
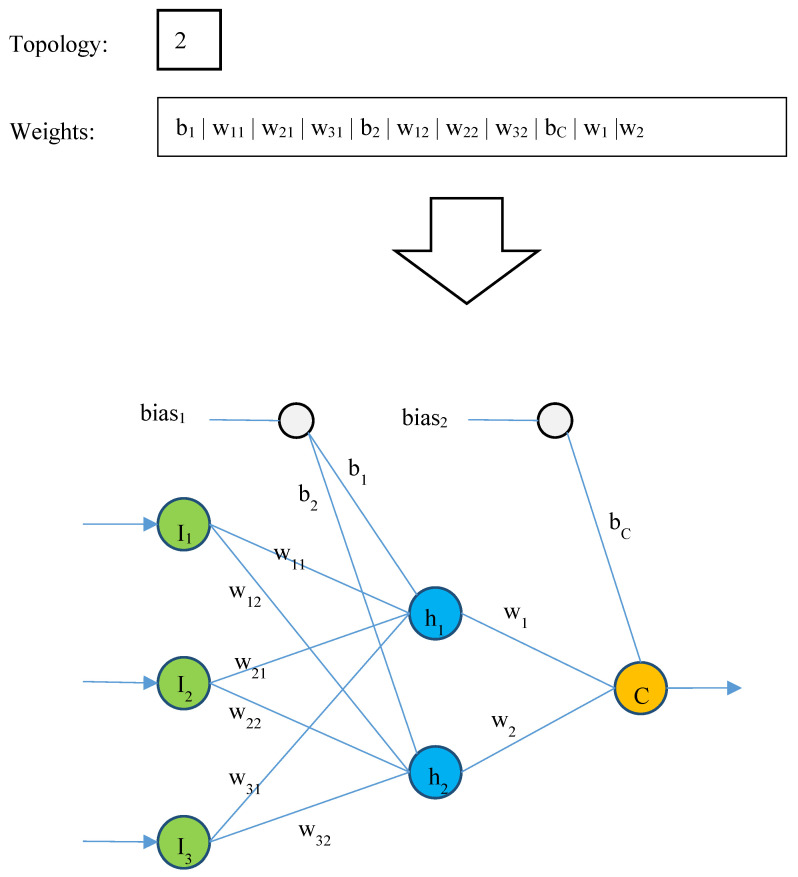
An example response vector for determining the topology and weight vector of a DBN.

Since the number of neurons in the DBN is determined in the first part of the solution vector, the length of the second part of the solution vector is determined based on that. For a DBN with *I* input neurons, *H* hidden neurons, and *P* output neurons, the length of the second part of the solution vector in the PSO algorithm is equal to H×(I+1)+P×(H+1). Figure 2 shows an example of a solution vector.

The network structure in the first section of the hypothetical solution vector, as shown in Figure 2, is a DBN with two neurons and a single hidden layer. In a similar vein, the second component of the solution vector in the process of assigning weights and biases to the generated DBN has a length of 11 if there are three input features and one output neuron. The obtained network’s simplified structure is shown in the bottom portion of Figure 2. The search boundaries for the second portion of the solution vector are likewise set to [−2, +2] throughout the PSO, while the first part of the solution vector is initialized randomly. In this way, each input weight value for the DBN’s bias and neuronal connections takes a value from this range. The neural network processes the training samples to produce outputs, the values of which are then compared to the real target values. These values are acquired by figuring out the deep belief neural network’s weights using the solution vector. The neural network’s performance and the written response’s optimality are then evaluated using the Mean Absolute Error (MAE) measure. As a result, the PSO algorithm’s fitness function is defined as follows:

(2) $$MAE=\frac{1}{N}\sum_{i=1}^{N}\left|T_{i}-Z_{i}\right|$$

where *T_i_* is the target value for the *i*th training sample and *N* is the number of training samples. Additionally, *Z_i_* is the DBN’s output for the *i*th training sample. Because of this, the suggested technique optimizes each DBN model using PSO, resulting in an ensemble system that may be used to forecast students’ academic success. Prior to doing this, the relevance of each DBN model’s output in the ensemble system’s output is ascertained using a reinforcement learning technique and assigned a weight value. The procedure is described in the section that follows.

#### 3.2.3. Weighting Learning Components in the Ensemble System Based on Reinforcement Learning

Using the weighted average is the last step in the suggested strategy for forecasting academic success. The aim of the averaging strategy is to increase algorithm learning accuracy as compared to the scenario in which each algorithm is applied independently. This method is referred to as ensemble learning or learning based on averaging. This approach uses a combination of various learning algorithms, and the average of the results is used to determine the system’s ultimate output. This technique has the potential to reduce prediction error. The value of each learning model’s output, however, can vary from the others due to variations in how well the models predict the target variable. To improve system accuracy, it may be more useful to weight each learning model’s output in order to assess how it affects the ensemble system’s ultimate output. Because of this, the ideal weight for each ensemble system component is determined using the reinforcement learning methodology in the suggested method. The suggested approach makes use of an LA as its reinforcement learning model.

In the present study, an LA model is developed for each learning model of the suggested ensemble system. This model is in charge of figuring out what the ideal weight value for each learning model is. As a result, there are three LAs used in the suggested strategy. According to its learning algorithm, each learning model’s random LA in this method simulates various weight allocation states. Following each evaluation, it raises the likelihood of appropriate weight allocation states and lowers the likelihood of inappropriate weight allocation states. In this stage, each learning model’s starting weight value is set to 1. Each DBN’s weight value is then modified using an LA in an iterative manner. The collection of options available to each LA constitutes the approach for adjusting the weight value of the associated DBN model. The notation for this set is $A=\left\{\alpha_{1},\alpha_{2},\ldots ,\alpha_{n}\right\}$. Every action in set A has a chance of being selected, and the set of probabilities is used to determine which action is chosen. To act, an automaton selects an action from set A, applies it to the environment (i.e., updates the DBN weight), and then waits for the environment (ensemble system) to assess it. The automaton then utilizes the environment’s reaction to determine what to do next. By modifying the likelihood of actions depending on reward and punishment parameters, the automaton learns which action (weight adjustment pattern) is best and should be selected with a greater probability throughout this process.

As previously mentioned, in the proposed method, an LA model is assigned to each DBN model present in the ensemble system. This model considers an action for different cases of weight change in its DBN output. In the proposed method, the set of actions of each LA is A={−0.1,−0.05,−0.01, 0, 0.01, 0.05, 0.1}. Therefore, the proposed method utilizes three LA models, each with *K* = 7 actions. In this study, *K* = 7 was chosen because it was a trade-off between exploration and exploitation for the number of actions in the LA’s action set. As discussed in (Sutton and Barto 2018), a larger action set in the LA would generally lead to exploring in the solution space to discover potentially better solutions, but would also require more computation, and hence, more iterations could be slower. On the other hand, a small number of actions may reduce the LA’s capacity to determine the best solution. From the experiments, we concluded that by setting *K* = 7, we can balance these factors for the LA to learn the weights optimally without much computation. Each of these LA models is responsible for determining the optimal weight for the corresponding DBN model.

The process of determining the optimal weight of DBN models using reinforcement learning is iterative, and its objective is to maximize the final detection accuracy by changing the weight values of the learning models of the ensemble system. At the start of the weighting process, all actions of each LA have an equal probability and are equal to $\frac{1}{K}$. The set of automaton probabilities is represented as $P=\left\{p_{1},p_{2},\ldots ,p_{K}\right\}$. The goal of the LA is to determine the probability value of each of the selectable actions, based on their optimal conditions. To update each of the values in the probability vector in the LA, first, the action with the highest probability value is selected (if the number of actions with maximum probability is more than one, one of these actions is randomly selected).

Once an LA chooses an action, the chosen action’s value is added to the DBN model’s existing weight value to create a new weight value for that learning model. The learning models in this iteration are weighted by repeating this procedure for all current LAs. Following the determination of each DBN model’s weight using the training data and the established weights, weighted averaging is used to determine the ensemble system’s output, and the accuracy of the system is computed for the established weights. The system’s correctness for the established weights is regarded as the environment’s reaction. Thus, after the environment’s reaction, the accuracy acquired is compared to the greatest accuracy in earlier iterations, and the action of updating each LA model’s probabilities is carried out based on the outcome of this comparison. Consequently, the following circumstances could materialize upon receipt of the environment’s reaction and comparison with the greatest accuracy in prior iterations:

- The set of LAs in the proposed system is able to select weight values that have increased detection accuracy and can help the system move toward global optimization if the accuracy in the current iteration for the currently determined weights (environment response) is higher than the highest accuracy in previous iterations. The set of activities that the LA chose for this cycle are thus regarded as the best options. In this instance, each LA uses the connection (current action *i* is examined) to enhance the likelihood of choosing the final action (Farahani et al. 2022):
  (3) $$p_{j}\left(k+1\right)=\left\{\begin{array}{cc} p_{j}\left(k\right)+a[1-p_{j}\left(k\right)] & j=i,\\ \left(1-a\right)p_{j}\left(k\right) & \forall \;j\neq i. \end{array}\right.$$
- If the accuracy in the current iteration is less than the highest accuracy in previous iterations, then the response generated in the recent cycle (and the weights selected by the LA) are considered to be non-optimal choices. In this case, each LA reduces the probability of selecting the last action using Equation (4) (Farahani et al. 2022).
  (4) $$p_{j}\left(k+1\right)=\left\{\begin{array}{cc} (1-b)p_{j}\left(k\right) & j=i,\\ \left(\frac{b}{K-1}\right)+\left(1-b\right)p_{j}\left(k\right) & \forall \;j\neq i. \end{array}\right.$$
  where the reward and punishment coefficients, respectively, are represented by the parameters *a* and *b*. These two parameters are assumed to be equal to 0.5 in the suggested procedure. Furthermore, *K* is the number of chosen actions in each automaton, and *k* is the discrete time index (the number of times probabilities are adjusted). The probability vector of every LA is updated after the application of the aforementioned criteria to each of the actions of the LA (individually for every DBN model). The procedures of selection, the assessment of the environment’s reaction, and updating the probability vector are carried out again from the first step once the LA models have been updated. This procedure keeps going until the algorithm’s iteration count hits the predefined threshold T. Consequently, the following are the phases of the suggested strategy for weighting DBN models using the reinforcement learning technique:
  1. Assign an LA to each DBN model in the ensemble system and set its initial weight to 1.
  2. For each LA, carry out the following steps:
    - Define the automaton actions as $A=\left\{-0.1,-0.05,-0.01,\;0,\;0.01,\;0.05,\;0.1\right\}$, each of which corresponds to the states of updating the current weight of the DBN model.
    - Set the automaton probabilities as P = {1/7, 1/7, 1/7, 1/7, 1/7, 1/7, 1/7}.
  3. Calculate the accuracy of the ensemble system based on unweighted averaging and store it in *X* (in this case, the weight of all DBN models is considered to be 1).
  4. Set the iteration counter to 1.
  5. For each LA, select the action with the highest probability in the P model of the automaton and update the weight value of the DBN model corresponding to it.
  6. Based on the weight values determined for each DBN model, perform weighted averaging and store the accuracy obtained in Y.
  7. If Y>X, then carry out the following steps:
    - Update the highest accuracy obtained to X = Y.
    - Store the weight values determined in the current iteration in W*.
    - Reward the actions selected by the LA in the current iteration using Equation (3).
  8. Otherwise, if Y≤X, then carry out the following steps:
    - Penalize the actions selected by the LA in the current iteration using Equation (4).
  9. Increase the iteration counter by one (iteration++).
  10. If iteration < T, then go to step 5; otherwise, go to the next step.
  11. Return the weight values stored in W* as the optimal weights of the DBN models of the ensemble system.

## 4. Research Findings

The cross-validation approach was used to conduct this research. Ten folds were employed in the cross-validation process, meaning that 10% of the data for each fold were used for testing and the remaining 90% for model training.

Three distinct modes were investigated in this research to see whether they might predict students’ academic success.

The first method is referred to as Proposed and is discussed in Section 3. Proposed (All Indications) is the option that makes use of all indications for prediction, except the first suggested stage. This makes it possible for us to look at how feature selection affects prediction accuracy. The weighted output combination step is skipped in the third mode, conventional ensemble, and predictions are made using the models’ basic average output. Additionally, the suggested approach is contrasted with studies conducted by Pallathadka et al. (2023), Lau et al. (2019), and Beckham et al. (2023). Beckham et al. (2023) presented a detailed description of a range of machine learning approaches for student performance prediction, which makes it a good benchmark. The study by Lau et al. (2019) concentrated on artificial neural networks, which are at the heart of our proposed ensemble, and the authors’ findings provided useful information on the feasibility of the approach. Pallathadka et al. (2023) explored the same set of machine learning algorithms and used them for the prediction of student performance, which gives a background to the current study. These works were chosen according to the goals of this research and the features used in the work for the prediction of student performance. In order to evaluate the quality of the selected studies, we compared the results of the studies in terms of accuracy, precision, and other parameters. We also assessed the reliability of the methods used in data collection, data preprocessing, and model evaluation. By focusing on studies that demonstrated competitive and high performance compared to previous work in their respective domains, we sought to compare our technique to state-of-the-art methods in student performance prediction. All of the mentioned approaches were implemented and evaluated using the same data.

The RMSE is a metric that calculates the average of the squares of the errors between predicted and actual values, resulting in a single number that highlights a model’s ability to predict continuous numeric outcomes. A way for representing root-mean-square error is as follows:

(5) $$RMSE=\sqrt{\frac{1}{N}\sum_{i=1}^{N}{\left(y_{i}-{\hat{y}}_{l}\right)}^{2}}$$

where *N* denotes the number of data samples. $Y_{i}$ represents the label vector of the ith sample, and ${\hat{y}}_{l}$ is the corresponding predictions of the sample using the proposed method.

MAPE (Mean Absolute Percentage Error) is a statistic for evaluating forecast accuracy. The following calculation calculates the average absolute percentage error between actual and predicted values:

(6) $$MAPE=\frac{1}{N}\sum_{i=1}^{N}\frac{\left|y_{i}-{\hat{y}}_{i}\right|}{y_{i}}$$

where $y_{i}$ is the actual value of the performance of students, ${\hat{y}}_{i}$ is the forecast value, and N is total number of observations.

**Figure 3:**
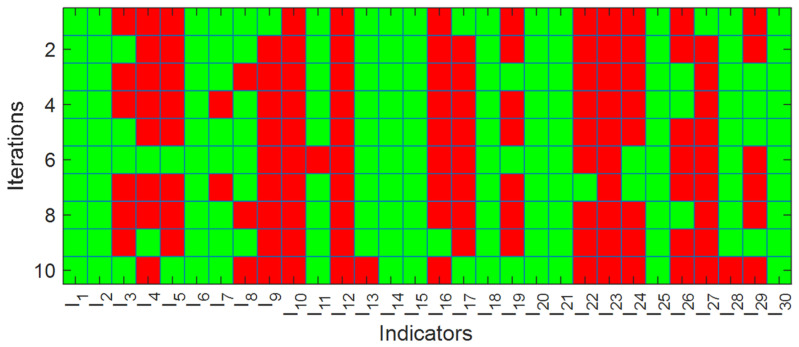
Details of selected features in each iteration of the experiments.

Figure 3 shows the rate of selecting each feature during various iterations. This figure demonstrates how qualities are selected over numerous cycles. This section has 30 horizontal suggestions and 10 vertical repetitions. The selection of attributes during various iterations is demonstrated as green boxes, while the red boxes show deleting an attribute during the feature selection process. characteristics chosen more than half of the time in iterations may be considered relevant and differentiated from other characteristics.

**Figure 4:**
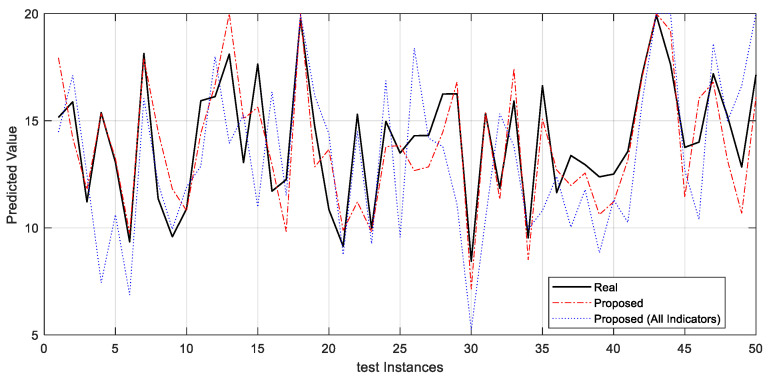
Real values of target variable versus values predicted by different algorithms.

Figure 4 depicts the projected values for student accomplishment, with the actual values given by the black line, the proposed technique marked by the red dashed line, and Proposed (All Indicators) represented by the blue dashed line. The strong resemblance between the lines of the recommended strategy and the observed values indicates the proposed technique’s commendable effectiveness when compared to the real case.

**Figure 5:**
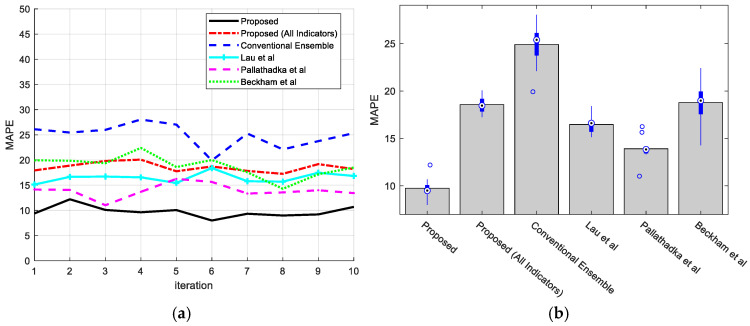
Performance evaluation of proposed method in comparison with the methods of Pallathadka et al. (2023), Lau et al. (2019), and Beckham et al. (2023) based on MAPE: (**a**) MAPE values in each iteration and (**b**) box plot of MAPE after 10 iterations.

Figure 5a shows the MAPE used to evaluate students’ prediction performance. The suggested technique has the lowest error value of 9.7, outperforming other comparable methods. MAPE was computed as the average percentage error between projected and actual values. This suggests that the suggested prediction approach aligns quite well with other methodologies. Figure 5b shows the error change intervals as boxplot graphs.

**Figure 6:**
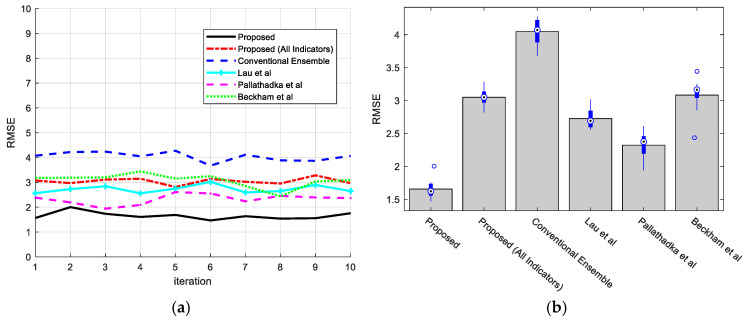
Performance evaluation of proposed method in comparison with the methods of Pallathadka et al. (2023), Lau et al. (2019), and Beckham et al. (2023) based on RMSE: (**a**) RMSE values in each iteration and (**b**) box plot of RMSE after 10 iterations.

Figure 6a,b show that positive qualities have decreased prediction errors over time, as indicated by the RMSE analysis and boxplots. Compared to past situations and other approaches, the suggested method consistently produces fewer squared errors, higher accuracy, and a lower RMSE, enhancing the chance of exact outcomes. These results reveal that the proposed methodology outperforms existing approaches in terms of dependability and accuracy in forecasting student performance, with a value of 1.6.

**Figure 7:**
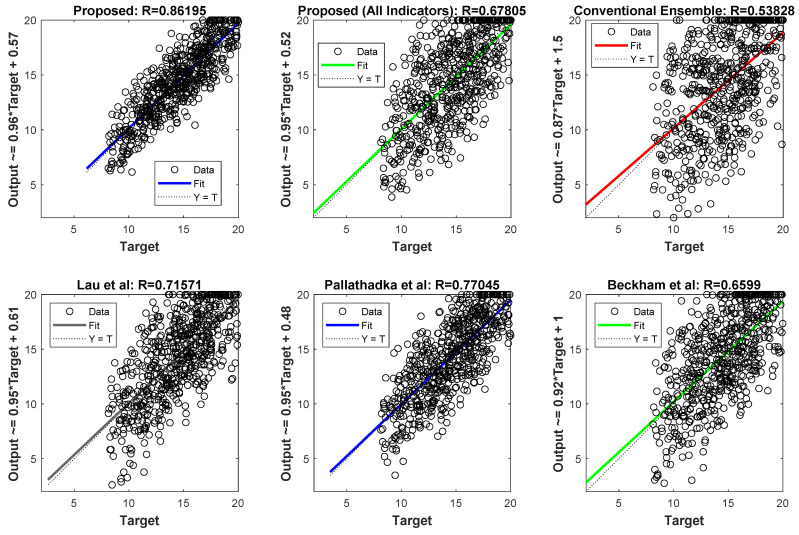
Regression plots of the proposed method and the methods of Pallathadka et al. (2023), Lau et al. (2019), and Beckham et al. (2023) for predicting the target variable.

The results of the students’ performance prediction are shown in Figure 7 as a linear regression plot, which graphically represents the correlation between the actual values and the values predicted by the suggested technique. Regression graphs make it abundantly evident that the results produced by the suggested approach are more consistent with the real outcomes. Each graph’s R value indicates the degree of correlation between the expected and actual values. With a prediction consistency of 0.86, the suggested method’s improved predictive capacity for student performance is shown by its higher R value when compared to other approaches. By using linear regression analysis, teachers may identify areas for growth, understand the elements that contribute to students’ performance, and customize interventions. This process allows for data-driven decision making and improves the quality of education for all students.

**Figure 8:**
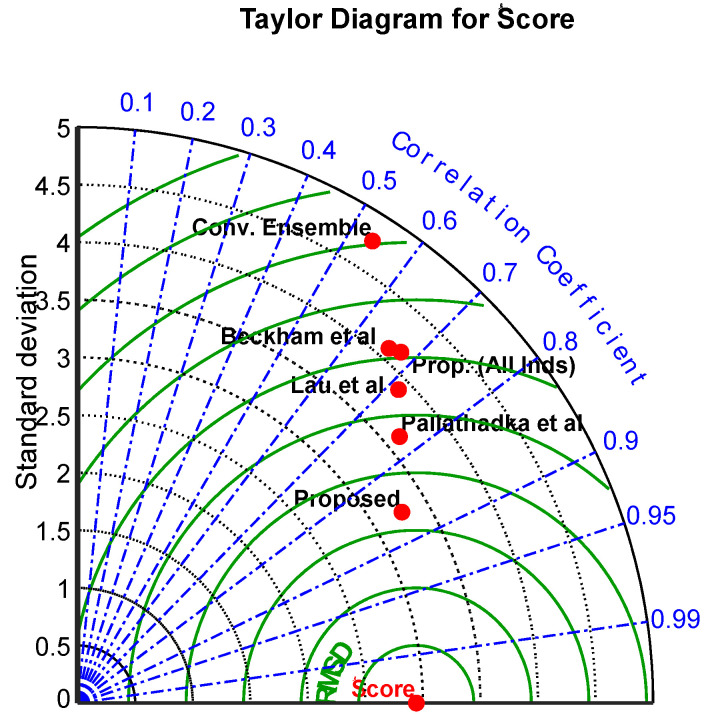
A Taylor diagram of the proposed method and the methods of Pallathadka et al. (2023), Lau et al. (2019), and Beckham et al. (2023) for predicting the target variable.

The examination of the following three distinct approaches is shown by the Taylor diagram in Figure 8: correlation, RMSE measure, and standard deviation. This study’s suggested strategy shows promise for achieving lower RMSE and standard deviation values with a greater correlation coefficient. These findings imply that the results produced by the suggested strategy are better than those produced by the other approaches. As a result, the Taylor diagram is an essential tool that helps people make judgments about predicting student performance and identifying areas that need improvement.

**Figure 9:**
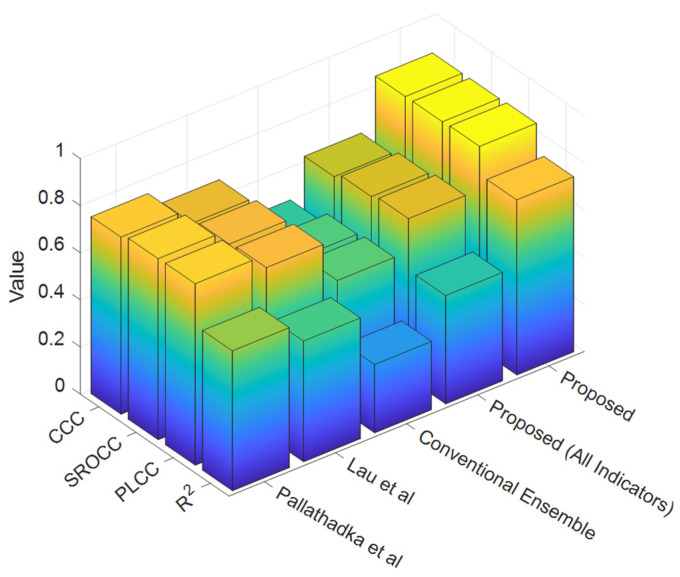
A performance comparison of the proposed method and the methods of Pallathadka et al. (2023), Lau et al. (2019), and Beckham et al. (2023) based on the criteria R^2^, PLCC, SROCC, and CCC.

Figure 9 depicts the measurements of PLCC, SROCC, CCC, and R-squared. The recommended technique for estimating student achievement took into account four key elements. Initially, the Pearson’s linear correlation coefficient (PLCC) criterion was used to evaluate the accuracy of the linear association between predicted and observed values. Subsequently, the Spearman’s rank-order correlation coefficient (SROCC) criteria, derived from Spearman’s ranking, were used to demonstrate a robust sensitivity to relative variations between the observed and forecasted values. The concordance correlation coefficient (CCC) criteria were used to merge correlation and conformance, therefore illustrating the level of agreement between expectations and reality. The concurrent use of these four indicators facilitated the assessment of the forecast’s comprehensive performance, ensuring the precision and reliability of the predictions. Ultimately, the R-squared criterion measures range between 0 and 1, where higher values indicate that the model has a greater ability to comprehend and predict product values. This methodology may be used to assess the precision and consistency of agricultural product forecasts, thereby enhancing the quality and reliability. PLCC is defined as follows:

(7) $$PLCC=\frac{\sum_{i=1}^{N}\left(S_{i}-\bar{S})(P_{i}-\bar{P}\right)}{\sqrt{\sum_{i=1}^{N}{\left(S_{i}-\bar{S}\right)}^{2}{\left(P_{i}-\bar{P}\right)}^{2}}}$$

where *n* represents the number of data points, $S_{i}\;$and $P_{i}\;$denote individual data points for the two variables, and $\bar{S\;}$ and $\bar{P}$ denote the means of the two variables.

The most common SROCC is

(8) $$SROCC=1-\frac{6\sum d_{i}^{2}}{n\;(n^{2}-1)}$$

where *n* is the number of data points, and $d_{i}$ represents the difference between the ranks of each corresponding pair of values.

To calculate CCC, use the following equation:

(9) $$CCC=\frac{2\sum_{i=1}^{d-1}\sum_{j=i+1}^{d}\sigma_{ij}}{\left(d-1\right)\sum_{i=1}^{d}\sigma_{i}^{2}+\sum_{i=1}^{d-1}\sum_{j=i+1}^{d}(\mu_{i-}\mu_{j}{)}^{2}}$$

where $\sigma_{i}^{2}$ and $\mu_{i}$ are the variance and mean of the measurements made by observer *i,* and $\sigma_{ij}$ is the covariance between the measurements from observers *i* and *j*.

(10) $$R^{2}=\frac{SSR}{SST}$$

(11) $$SSR=\sum_{i}({\hat{y}}_{i}-\bar{y}{)}^{2}$$

(12) $$SST=\sum_{i}(y_{i}-\bar{y}{)}^{2}$$

where SSR is the sum of the squared regression, also known as the variation explained by the model; SST is the total variation in the data, also known as the sum of the squared total; $y_{i}\;$is the *y* value for observation *i*; $\bar{y}\;$is the mean of the y value; and $\hat{y}$ is the predicted value of y for observation *i*.

Table 2 presents a concise overview of the outcomes derived from the assessments. For further evaluation of the proposed method, a comparison with the simple linear regression model was made. A summary of this implementation is given in Table 2. The linear regression model gave an RMSE of 6.7911 and an MAPE of 41.0622. In contrast, the proposed model yielded much lower RMSE and MAPE values, proving the better predictive capability of this model.

The suggested technique regularly surpasses the other comparison methods in terms of all examined parameters, demonstrating improved prediction accuracy, stronger correlations, and better agreement between anticipated and actual values.

The suggested technique typically outperforms the conventional ensemble model, as well as the models of Lau et al. (2019), Pallathadka et al. (2023), and Beckham et al. (2023), and the linear regression model, in terms of greater RMSE and MAPE values, and lower R^2^, PLCC, SROCC, and CCC values.

## 5. Discussion, Limitations, and Future Directions

This section presents a discussion about the performance of the proposed method and its implications for educational practices. Finally, after acknowledging the limitations of this study, several suggestions for future research are provided.

### 5.1. Performance of the Proposed Model

The deep ensemble learning model proposed in this study outperformed the other methods in the prediction of student academic performance. As highlighted in Table 2, the model was able to make predictions with an RMSE of 1.6562 and an MAPE of 9.7532, which was much better than the null model (conventional ensemble with RMSE of 4.0467 and MAPE of 24.8950) and the benchmark models of Lau et al. (2019), Pallathadka et al. (2023), and Beckham et al. (2023).

The Relief and MRMR methods of feature selection were significant in improving the performance of the model. These techniques helped in reducing noise and enhanced predictive accuracy by only considering features that are most relevant to the model. This is seen especially in comparing the performance of the proposed model and “Proposed (All Indicators)”, which used all the features without feature selection.

In addition, the weighted averaging scheme helped the model do well because it allowed for the integration of the predictions of various DBNs. This approach was able to take advantage of the different strengths of the various components, hence providing more accurate predictions. The enhanced performance demonstrated by the proposed model affirms the significance of feature selection, as well as ensemble approaches, in enhancing the prediction of student performance.

### 5.2. Implications for Educational Practice

The accurate prediction of student performance has a lot of implications for institutions of learning. If students who are in danger of experiencing academic challenges are first recognized, measures can be put in place to boost the students’ achievement and retention. The proposed model can also be used in decisions concerning the distribution of available resources including academic advising, which can enhance the efficiency of utilization of educational resources and the overall performance of students.

### 5.3. Limitations and Future Research

While these results are promising, there are some limitations that need to be discussed in this section. The applicability of the results might be limited by the specification of the target population that the data were gathered from. Because the utilized dataset includes data from a specific region and a limited age range. It is possible to continue the study of the effectiveness of the proposed model in other educational settings and with other students.

Also, this study was confined to a static prediction model. Including the longitudinal data to monitor the students’ performance over time in the course of their studies could be useful to analyze the changes in their performance and the effectiveness of the model. Exploring the application of other techniques related to explainable AI to further explain the workings of the model and to make the model more interpretable would also be a good area of future work.

In addition, the effect of various feature selection techniques and ensemble architectures on model performance is an area that deserves further research. In this way, by stating the limitations of the current research and by outlining the possible avenues for the future development of the field of student performance prediction, it is possible to contribute to the further development of the field.

## 6. Conclusions

The usefulness of the suggested strategy for forecasting student performance, which combines feature ranking and deep learning models, has been proven to improve the accuracy of academic performance projections. This approach utilizes Deep Belief Networks, which are improved by particle swarm optimization, and autoencoders to alter weight values. It provides a strong foundation for studying and comprehending the intricate correlations between many indicators and student performance. Our experimental findings, characterized by an RMSE of 0.7 and an MAPE of 4.2, underscore the considerable potential of this technique to enhance the accuracy of student performance prediction. This not only helps educational institutions in devising more efficient approaches to improve academic achievements, but also aids in identifying students who are in danger of experiencing academic challenges, enabling early interventions to prevent them from dropping out. The technique described in this work establishes a strong basis for future study and development in the area of educational data analytics, as technology advances and produces more advanced learning aids.

## Figures and Tables

**Table 1:** The specifications of the database used in this research.

ID	Title	Type
I1	Place of Study	Nominal (Discrete)
I2	Gender	Nominal (Discrete)
I3	Age	Continuous (Numeric)
I4	Residence Status	Nominal (Discrete)
I5	Number of Family Members	Discrete (Numeric)
I6	Parents’ Marital Status	Nominal (Discrete)
I7	Mother’s Education Level	Ordinal (Ranked)
I8	Father’s Education Level	Ordinal (Ranked)
I9	Mother’s Employment Status	Nominal (Discrete)
I10	Father’s Employment Status	Nominal (Discrete)
I11	Reason for Choosing Place of Study	Nominal (Discrete)
I12	Legal Guardian	Nominal (Discrete)
I13	Distance from Home to School	Continuous (Numeric)
I14	Weekly Study Time	Continuous (Numeric)
I15	Average Grades in Previous Term	Continuous (Numeric)
I16	Scholarship Status	Nominal (Discrete)
I17	Parental Financial Support for Education	Nominal (Discrete)
I18	Use of Extracurricular Classes	Nominal (Discrete)
I19	Participation in Extracurricular Activities	Nominal (Discrete)
I20	Participation in Scientific Competitions	Nominal (Discrete)
I21	Desire to Continue Education	Nominal (Discrete)
I22	Internet Access at Residence	Nominal (Discrete)
I23	Emotional Relationship Status	Nominal (Discrete)
I24	Quality of Relationship with Family Members	Ordinal (Ranked)
I25	Amount of Free Time After Classes	Continuous (Numeric)
I26	Interaction with Classmates Outside of School	Nominal (Discrete)
I27	Alcohol Consumption During Week	Nominal (Discrete)
I28	Alcohol Consumption During Weekend	Nominal (Discrete)
I29	Current Health Status	Ordinal (Ranked)
I30	Number of Absences in Class	Continuous (Numeric)
-	Student’s Final Average Grades	Continuous (Numeric)

**Table 2:** Summary of results obtained from evaluations.

Methods	RMSE	MAPE	R^2^	PLCC	SROCC	CCC
Proposed	1.6562	9.7532	0.7430	0.8619	0.8582	0.8571
Proposed (All Indicators)	3.0489	18.5643	0.4597	0.6780	0.6634	0.6397
Conventional Ensemble	4.0467	24.8950	0.2897	0.5383	0.5279	0.4788
Lau et al. (2019)	2.7256	16.4662	0.5122	0.7157	0.7074	0.6878
Pallathadka et al. (2023)	2.3228	13.9115	0.5936	0.7704	0.7685	0.7518
Beckham et al. (2023)	3.0828	18.7673	0.4355	0.6599	0.6485	0.6245
Linear Regression	6.7911	41.0622	0.0447	0.2115	0.2113	0.1513

## Data Availability

The original contributions presented in the study are included in the article, further inquiries can be directed to the corresponding author.

## References

[B1-jintelligence-12-00124] Agnafors Sara, Barmark Mimmi, Sydsjö Gunilla (2021). Mental health and academic performance: A study on selection and causation effects from childhood to early adulthood. Social Psychiatry and Psychiatric Epidemiology.

[B2-jintelligence-12-00124] Ahmed Shams Forruque, Alam M. Sakib Bin, Hassan Maruf, Rozbu Mahtabin Rodela, Ishtiak Taoseef, Rafa Nazifa, Mofijur M., Ali A. B. M. Shawkat, Gandomi Amir H. (2023). Deep learning modelling techniques: Current progress, applications, advantages, and challenges. Artificial Intelligence Review.

[B3-jintelligence-12-00124] Alyahyan Eyman, Düştegör Dilek (2020). Predicting academic success in higher education: Literature review and best practices. International Journal of Educational Technology in Higher Education.

[B4-jintelligence-12-00124] Antonopoulou Marina, Mantzorou Maria, Serdari Aspasia, Bonotis Konstantinos, Vasios Giorgos, Pavlidou Eleni, Trifonos Christina, Vadikolias Konstantinos, Petridis Dimitris, Giaginis Constantinos (2020). Evaluating Mediterranean diet adherence in university student populations: Does this dietary pattern affect students’ academic performance and mental health?. The International Journal of Health Planning and Management.

[B5-jintelligence-12-00124] Asaad Renas Rajab, Ali Rasan Ismael (2019). Back Propagation Neural Network (BPNN) and sigmoid activation function in multi-layer networks. Academic Journal of Nawroz University.

[B6-jintelligence-12-00124] Asadollahfardi Gholamreza (2015). Artificial neural network. Water Quality Management: Assessment and Interpretation.

[B7-jintelligence-12-00124] Asselman Amal, Khaldi Mohamed, Aammou Souhaib (2023). Enhancing the prediction of student performance based on the machine learning XGBoost algorithm. Interactive Learning Environments.

[B8-jintelligence-12-00124] Baker Ryan S. D., Corbett Albert T., Aleven Vincent (2008). More accurate student modeling through contextual estimation of slip and guess probabilities in bayesian knowledge tracing. Paper presented at Intelligent Tutoring Systems: 9th International Conference, ITS 2008.

[B9-jintelligence-12-00124] Beckham Nicholas Robert, Akeh Limas Jaya, Mitaart Giodio Nathanael Pratama, Moniaga Jurike V. (2023). Determining factors that affect student performance using various machine learning methods. Procedia Computer Science.

[B10-jintelligence-12-00124] Bendangnuksung Prabu P. (2018). Students’ performance prediction using deep neural network. International Journal of Applied Engineering Research.

[B11-jintelligence-12-00124] Bruffaerts Ronny, Mortier Philippe, Kiekens Glenn, Auerbach Randy P., Cuijpers Pim, Demyttenaere Koen, Green Jennifer G., Nock Matthew K., Kessler Ronald C. (2018). Mental health problems in college freshmen: Prevalence and academic functioning. Journal of Affective Disorders.

[B12-jintelligence-12-00124] Corbett Albert T., Anderson John R. (1994). Knowledge tracing: Modeling the acquisition of procedural knowledge. User Modeling and User-Adapted Interaction.

[B13-jintelligence-12-00124] Divyabharathi Y., Someswari P. (2018). A Framework for Student Academic Performance Using Naïve Bayes Classification Technique. JAET.

[B14-jintelligence-12-00124] Farahani Mansoureh Ghiasabadi, Torkestani Javad Akbari, Rahmani Mohsen (2022). Adaptive personalized recommender system using learning automata and items clustering. Information Systems.

[B15-jintelligence-12-00124] Foli Sophia Korkor (2018). Backward Elimination Algorithm for High Dimensional Variable Screening. Master’s thesis.

[B16-jintelligence-12-00124] Francis Bindhia K., Babu Suvanam Sasidhar (2019). Predicting academic performance of students using a hybrid data mining approach. Journal of Medical Systems.

[B17-jintelligence-12-00124] Gad Ahmed G. (2022). Particle swarm optimization algorithm and its applications: A systematic review. Archives of Computational Methods in Engineering.

[B18-jintelligence-12-00124] Gamao Ariel O., Gerardo Bobby D. (2019). Prediction-based model for student dropouts using modified mutated firefly algorithm. International Journal of Advanced Trends in Computer Science and Engineering.

[B19-jintelligence-12-00124] Gil Jay S., Delima Allemar Jhone P., Vilchez Ramcis N. (2020). Predicting students’ dropout indicators in public school using data mining approaches. International Journal of Advanced Trends in Computer Science and Engineering.

[B20-jintelligence-12-00124] Gimenez Gregorio, Martín-Oro Ángel, Sanaú Jaime (2018). The effect of districts’ social development on student performance. Studies in Educational Evaluation.

[B21-jintelligence-12-00124] Gong Yue, Beck Joseph E., Heffernan Neil T. (2011). How to construct more accurate student models: Comparing and optimizing knowledge tracing and performance factor analysis. International Journal of Artificial Intelligence in Education.

[B22-jintelligence-12-00124] González-Brenes Jose, Huang Yun, Brusilovsky Peter (2014). General features in knowledge tracing to model multiple subskills, temporal item response theory, and expert knowledge. Paper presented at The 7th International Conference on Educational Data Mining.

[B23-jintelligence-12-00124] Guan Li (2023). Evaluating teaching quality in colleges using combination of artificial neural networks (ANNs) and black hole optimization (BHO). Heliyon.

[B24-jintelligence-12-00124] Gunn Heather J., Rezvan Panteha Hayati, Fernández M. Isabel, Comulada W. Scott (2023). How to apply variable selection machine learning algorithms with multiply imputed data: A missing discussion. Psychological Methods.

[B25-jintelligence-12-00124] Gütter Jonas, Kruspe Anna, Zhu Xiao Xiang, Niebling Julia (2022). Impact of training set size on the ability of deep neural networks to deal with omission noise. Frontiers in Remote Sensing.

[B26-jintelligence-12-00124] Hasan Raza, Palaniappan Sellappan, Raziff Abdul Rafiez Abdul, Mahmood Salman, Sarker Kamal Uddin (2018). Student academic performance prediction by using decision tree algorithm. Paper presented at 2018 4th International Conference on Computer and Information Sciences (ICCOINS).

[B27-jintelligence-12-00124] Hasan Raza, Palaniappan Sellappan, Mahmood Salman, Abbas Ali, Sarker Kamal Uddin, Sattar Mian Usman (2020). Predicting student performance in higher educational institutions using video learning analytics and data mining techniques. Applied Sciences.

[B28-jintelligence-12-00124] Hernández Juan Antonio Caballero, Alberico Antonio B., Duarte Manuel Palomo, Dellatorre Pablo, Reinoso Antonio J., Beardo Juan Manuel D. (2020). Teamwork assessment in collaborative projects through process mining techniques. The International Journal of Engineering Education.

[B29-jintelligence-12-00124] Jo Insik, Lee Sangbum, Oh Sejong (2019). Improved measures of redundancy and relevance for mRMR feature selection. Computers.

[B30-jintelligence-12-00124] Lau Erwin T., Sun Licheng, Yang Qingping (2019). Modelling, prediction and classification of student academic performance using artificial neural networks. SN Applied Sciences.

[B31-jintelligence-12-00124] Mengash Hanan A. (2020). Using data mining techniques to predict student performance to support decision making in university admission systems. IEEE Access.

[B32-jintelligence-12-00124] Minn Sein, Yu Yi, Desmarais Michel C., Zhu Feida, Vie Jill-Jenn (2018). Deep knowledge tracing and dynamic student classification for knowledge tracing. Paper presented at 2018 IEEE International Conference on Data Mining (ICDM).

[B33-jintelligence-12-00124] Mustapha Ishamuddin, Van N. T., Shahverdi Masoumeh, Qureshi Muhammad Imran, Khan Nohman (2021). Effectiveness of digital technology in education during COVID-19 pandemic. A bibliometric analysis. International Journal of Interactive Mobile Technologies (iJIM).

[B34-jintelligence-12-00124] Nijman Swj W. J., Leeuwenberg A. M., Beekers I., Verkouter I., Jacobs J. J. L., Bots M. L., Asselbergs F. W., Moons Kgm G. M., Debray Tpa P. A. (2022). Missing data is poorly handled and reported in prediction model studies using machine learning: A literature review. Journal of Clinical Epidemiology.

[B35-jintelligence-12-00124] Pallathadka Harikumar, Wenda Alex, Ramirez-Asís Edwin, Asís-López Maximiliano, Flores-Albornoz Judith, Phasinam Khongdet (2023). Classification and prediction of student performance data using various machine learning algorithms. Materials today: Proceedings.

[B36-jintelligence-12-00124] Palomo-Duarte Manuel, Dodero Juan Manuel, Medina-Bulo Inmaculada, Rodríguez-Posada Emilio J., Ruiz-Rube Iván (2014). Assessment of collaborative learning experiences by graphical analysis of wiki contributions. Interactive Learning Environments.

[B37-jintelligence-12-00124] Pelánek Radek (2017). Bayesian knowledge tracing, logistic models, and beyond: An overview of learner modeling techniques. User Modeling and User-Adapted Interaction.

[B38-jintelligence-12-00124] Pisner Derek A., Schnyer David M. (2020). Support vector machine. Machine Learning.

[B39-jintelligence-12-00124] Priyanka, Kumar Dharmender (2020). Decision tree classifier: A detailed survey. International Journal of Information and Decision Sciences.

[B40-jintelligence-12-00124] Raihana Z., Farah Nabilah A. M. (2018). Classification of students based on quality of life and academic performance by using support vector machine. Journal of Academia.

[B41-jintelligence-12-00124] Ramaphosa Khokhoni Innocentia Mpho, Zuva Tranos, Kwuimi Raoul (2018). Educational data mining to improve learner performance in Gauteng primary schools. Paper presented at 2018 International Conference on Advances in Big Data, Computing and Data Communication Systems (icABCD).

[B42-jintelligence-12-00124] Romero Cristóbal, Ventura Sebastián (2020). Educational data mining and learning analytics: An updated survey. Wiley Interdisciplinary Reviews: Data Mining and Knowledge Discovery.

[B43-jintelligence-12-00124] Sapare Naveen S., Beelagi Sahana M. (2021). Comparison study of Regression Models for the prediction of post-Graduation admissions using Machine Learning Techniques. Paper presented at 2021 11th International Conference on Cloud Computing, Data Science & Engineering (Confluence).

[B44-jintelligence-12-00124] Sarwat Samina, Ullah Naeem, Sadiq Saima, Saleem Robina, Umer Muhammad, Eshmawi Ala’ Abdulmajid, Mohamed Abdullah, Ashraf Imran (2022). Predicting students’ academic performance with conditional generative adversarial network and deep SVM. Sensors.

[B45-jintelligence-12-00124] Singh Randhir, Pal Saurabh (2020). Application of machine LearningAlgorithms to predict students performance. International Journal of Advanced Science andTechnology.

[B46-jintelligence-12-00124] Sutton Richard S., Barto Andrew G. (2018). Reinforcement Learning: An Introduction.

[B47-jintelligence-12-00124] Tsiakmaki Maria, Kostopoulos Georgios, Kotsiantis Sotiris, Ragos Omiros (2020). Transfer learning from deep neural networks for predicting student performance. Applied Sciences.

[B48-jintelligence-12-00124] Urbanowicz Ryan J., Meeker Melissa, Cava William La, Olson Randal S., Moore Jason H. (2018). Relief-based feature selection: Introduction and review. Journal of Biomedical Informatics.

[B49-jintelligence-12-00124] Waheed Hajra, Hassan Saeed-Ul, Aljohani Naif Radi, Hardman Julie, Alelyani Salem, Nawaz Raheel (2020). Predicting academic performance of students from VLE big data using deep learning models. Computers in Human behavior.

[B50-jintelligence-12-00124] Wickramasinghe Indika, Kalutarage Harsha (2021). Naive Bayes: Applications, variations and vulnerabilities: A review of literature with code snippets for implementation. Soft Computing.

[B51-jintelligence-12-00124] Xiong Xiaolu, Zhao Siyuan, Inwegen Eric G. Van, Beck Joseph E. (2016). Going deeper with deep knowledge tracing. Paper presented at the International Conference on Educational Data Mining (EDM).

[B52-jintelligence-12-00124] Yu Hao, Wilamowski Bogdan M. (2018). Levenberg–marquardt training. Intelligent Systems.

[B53-jintelligence-12-00124] Zorić Alisa Bilal (2019). Predicting Students’ Success Using Neural Network. Paper presented at 2019 ENTRENOVA Conference Proceedings.

